# Sjögren’s Disease and Oral Health: A Genetic Instrumental Variable Analysis

**DOI:** 10.1177/00220345231218903

**Published:** 2024-01-29

**Authors:** S.L. Reckelkamm, Z. Alayash, B. Holtfreter, M. Nolde, S.E. Baumeister

**Affiliations:** 1 Institute of Health Services Research in Dentistry, University of Münster, Münster, Germany; 2 Clinic for Periodontology and Conservative Dentistry, University of Münster, Münster, Germany; 3 Department of Restorative Dentistry, Periodontology, Endodontology, and Preventive and Pediatric Dentistry, University Medicine Greifswald, Greifswald, Germany

**Keywords:** dental caries, periodontitis, Mendelian randomization analysis, saliva, immunity, genetic epidemiology

## Abstract

Epidemiological studies have consistently shown that Sjögren’s disease (SjD) increases the risk of dental caries. Despite similar evidence indicating an elevated risk of periodontitis, SjD remains a disputed risk factor for this disease. The risk of bias in observational research is a major impediment to confirming this link. Within an instrumental variable framework, genetic variants associated with a risk factor can be used to proxy its effect on an outcome while avoiding common sources of observational study bias. In this study, we leveraged an instrumental variable approach to investigate whether SjD affects the risk of caries and periodontitis. A total of 57 genetic variants strongly associated with SjD were identified from a genome-wide association study of 2,247 European descent cases and 332,115 controls. We tested for associations of these genetic instruments with caries (measured as the number of decayed, missing, and filled surfaces in 26,792 individuals) and periodontitis (17,353 clinical periodontitis cases and 28,210 European controls). Several sensitivity analyses were used to further validate the primary inverse variance weighted (IVW) estimate. IVW analysis revealed an adverse effect of SjD on caries (β = 0.039, *P* = 6.3e-16) and periodontitis (odds ratio = 1.033, *P* = 2.3e-05). Sensitivity analyses, conducted to assess the robustness to potential violations of instrumental variable assumptions, further support these findings. Our results showed that SjD has a detrimental effect on caries and also suggest that SjD promotes periodontitis.

## Introduction

Sjögren’s disease (SjD) ranks among the most prevalent autoimmune diseases and is characterized by destructive inflammatory processes in exocrine glands, particularly the salivary and lacrimal glands (Brito-Zerón et al. 2016). Estimates of its prevalence range from 0.01% to 0.72% depending on classification criteria, and a significant number of unreported cases are suspected (Maldini et al. 2014). Women are disproportionately affected, rendering SjD one of the most unequally distributed autoimmune disorders (Ramos-Casals et al. 2015). The etiology of the underlying auto-reactivity is unknown, but it is thought to be the result of a complex genetic background interplaying with environmental influences (Brito-Zerón et al. 2016).

The slow, cumulative deterioration of exocrine glands causes extreme dryness of the eyes and mouth (Moutsopoulos 1994). Patients commonly report difficulties in swallowing, altered taste perception, and burning mouth syndrome as their primary oral health concerns. Clinically, the oral mucosa appears dry, erythematous, and sticky (Carr et al. 2012). Additionally, SjD increases the risk of dental caries, which frequently affects sites that are usually resistant to decay, like the cervical regions and smooth surfaces of the teeth (Pedersen et al. 2005; Carr et al. 2012). Moreover, numerous studies have reported a higher prevalence of periodontal disease among individuals with SjD (Carr et al. 2012; Lin et al. 2019; Yang, Pang, et al. 2022).

Dental caries is a multifactorial disease in which acidic by-products of bacterial carbohydrate metabolism decompose dental hard tissues (Selwitz et al. 2007). Periodontitis is a complex microbially associated chronic inflammatory disease of the tissues surrounding the teeth. Both conditions rank among the most prevalent chronic diseases globally and are the primary causes of tooth loss (Kassebaum et al. 2017). The detrimental effect of SjD on caries susceptibility is generally acknowledged in the literature (Carr et al. 2012). However, the effect of SjD on periodontitis remains strongly debated (see Fig.) (Lin et al. 2019; Maarse et al. 2019; Yang, Pang, et al. 2022; Gheorghe et al. 2023). Aside from a less clear link between the cardinal symptom of xerostomia and periodontal health, this is primarily due to 2 well-known problems of conventional observational studies: confounding and reverse causation (Maarse et al. 2019; Yang, Pang, et al. 2022; Gheorghe et al. 2023). Given the intricate etiology of both diseases and their shared association with polyautoimmunity, the presence of (unobserved) confounding factors is suspected (Kollert and Fisher 2020; Hajishengallis and Chavakis 2021). The chronological ordering is further blurred by the late detection of SjD (usually in the fourth or fifth decade of life), which occurs only after serious complaints emerge (Patel and Shahane 2014). Periodontal changes, although presumably attributable to SjD, may thus appear ahead of the condition being officially diagnosed. Fortunately, genetic instrumental variable (IV) analysis is a potent methodological solution in medical research to address these issues (Maciejewski and Brookhart 2019). Following Mendel’s laws of inheritance, genetic variations are randomly inherited, providing balance in observed and unobserved confounders. Moreover, as these variations occur at conception, long before the onset of either disease, the temporal sequence remains unambiguous (Davies et al. 2018).

**Figure.**
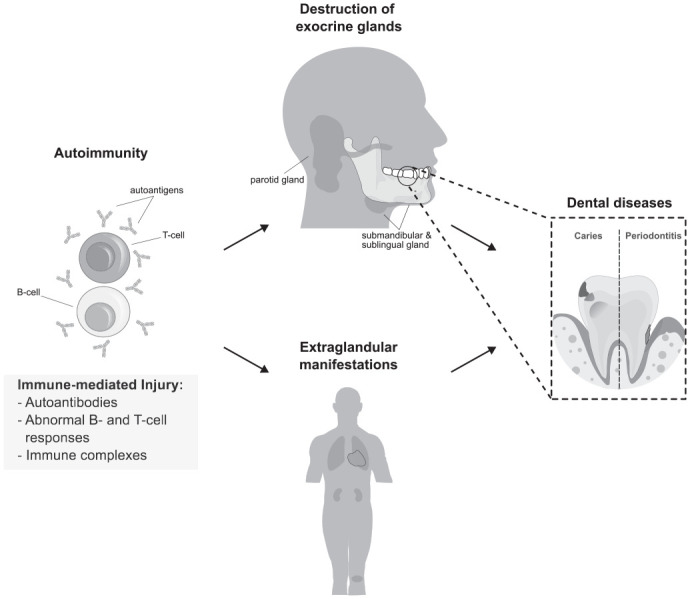
Pathophysiologic basis and supposed mechanisms of Sjögren’s disease (SjD). This schematic provides a brief overview of SjD pathogenesis. SjD is a chronic inflammatory condition characterized by gradual loss of function in the lacrimal and salivary glands, leading to the distinctive sicca symptoms (dry eyes and dry mouth). Additional manifestations of the disease include dryness of skin and other mucosal surfaces. Systematic manifestations encompass conditions like arthritis, nephritis, pneumonitis, and vasculitis (Mavragani 2017). The pathogenesis of SjD is conceptualized as a multistep process triggered by environmental factors, most likely of viral origin, in a genetically predisposed individual. The initial stimuli set the innate immune system in motion, but the ongoing autoimmune process requires perpetual interplay between the innate and adaptive immune systems. This results in autoreactive B- and T-cell responses, the production of autoantibodies, and the chronic inflammation of salivary and lacrimal glands, as well as other tissues. This inflammation eventually leads to the loss of physiological glandular function. Extraglandular manifestations may result from autoimmune exocrinopathy similar to that in the salivary glands, immune complex deposition, and/or extranodal lymphoproliferation. Sustained stimulation of B cells may also promote lymphomagenesis in susceptible individuals (Mavragani 2017; Mavragani and Moutsopoulos 2020). Oral dryness increases the risk of infection, reduces salivary flow, impairs rinsing function, and hinders tooth remineralization. While an increased susceptibility to dental caries is well recognized, the direct link between SjD and periodontitis is a subject of controversy and requires further confirmation (Maarse et al. 2019; Yang, Pang, et al. 2022; Gheorghe et al. 2023). Given the exposed anatomical position of the teeth, it can be anticipated that changes in saliva flow or its composition have the greatest impact on dental hard tissues. However, other processes connected to the extraglandular immune-mediated damage mentioned previously may further affect the periodontium. This study investigates the potential impact of SjD on both conditions, irrespective of individual pathways.

In this study, we utilize such an IV framework to 1) replicate the known effect of SjD on dental caries and 2) reject the null hypothesis of no effect of SjD on periodontitis.

## Materials and Methods

We leveraged genetic variations randomly allocated at conception to elucidate causal relationships between a risk factor and an outcome of interest. These genetic variations in the form of single nucleotide polymorphisms (SNPs) were extracted from published genome-wide association studies (GWAS) of European populations and genetic studies identifying variants associated with a specific phenotypic trait (Davies et al. 2018). This study has been conducted in accordance with Strengthening the Reporting of Observational Studies in Epidemiology using Mendelian Randomization (STROBE-MR) recommendations (Skrivankova et al. 2021).

### Data Sources

Association estimates of SNPs for SjD were derived from the FinnGen project encompassing 2,247 diagnosed cases and 332,115 controls (Kurki et al. 2022). Genetic associations for dental caries (measured as the decayed, missing, and filled surfaces [DMFS] index in 26,792 individuals) and periodontitis (17,353 periodontitis cases and 28,210 controls defined by either the Centers for Disease Control and Prevention/American Academy of Periodontology [CDC/AAP] classification or the Community Periodontal Index [CPI] case definition) were derived from the GeneLifestyle Interactions in Dental Endpoints (GLIDE) consortium (see Table 1) (Shungin et al. 2019).

**Table 1.** Description of Genome-Wide Association Studies Used for Each Phenotype.

Phenotype	No. of Participants	First Author (Year)	PMID	Data Access Link
Sjögren’s disease	2,247 cases, 332,115 controls	Kurki (2022)	36653562	https://www.finngen.fi/en/access_results
DMFS index	26,792 individuals	Shungin (2019)	31235808	https://data.bris.ac.uk/data/dataset/2j2rqgzedxlq02oqbb4vmycnc2
Periodontitis	17,353 cases, 28,210 controls			

DMFS, decayed, missing, and filled surfaces; PMID, PubMed identifier.

### Selection of Instrumental Variables

We selected SNPs as instruments when they surpassed the genome-wide significance threshold (*P* < 5 × 10−8) and a linkage disequilibrium *r*² of 0.1 with a 10,000-kb window. We further estimated the *F* statistics and the phenotypic variance collectively explained by all instruments as indicators of instrument strength. *F* statistics >10 were considered sufficient to rule out weak instrument bias (Burgess et al. 2019).

### Statistical Analysis

Logically, if SNPs, which serve as valid proxies for SjD, are associated with a certain oral health outcome, it strongly suggests that SjD exhibits an impact on that specific characteristic, in our case caries or periodontitis. This logical conclusion serves as the guiding principle for subsequent IV investigations (Fu and Kim 2021). As this study differs from conventional observational studies and some readers may not be familiar with the approach used, we would like to refer to outstanding works on (genetic) instrumental variable analysis and causal language for a deeper understanding of these concepts (Pingault et al. 2018; Fu and Kim 2021; Listl et al. 2022). In the primary analysis, Wald ratios for each SNP were combined using inverse variance weighted (IVW) meta-analysis, resulting in an overall causal effect estimate of SjD on the dental traits (Burgess et al. 2019). The primary goal of this analysis is to reject the null hypothesis and estimate the direction of the effect (positive or negative) (Sheehan and Didelez 2020). For further illustration, we converted the corresponding estimate into more interpretable units. In terms of DMFS, this means a back transformation of βs and accompanying confidence intervals (CIs) into “number of affected tooth surfaces.” According to the formula published by the authors of the outcome GWAS:

$$\mathrm{Affected}\;\mathrm{tooth}\;\mathrm{surfaces}\;(\mathrm{DMFS})\;=\;\beta_{xy}\times 19.87$$

A corresponding increase by 1-unit change in the transformed DMFS score equates to an increase of 19.87 affected surfaces (Shungin et al. 2019; Dodhia et al. 2020). The obtained effect estimates for periodontitis represent log odds ratios (ORs). In accordance with the binary exposure, these were converted into interpretable ORs as follows:

$$\mathrm{Causal}\;\mathrm{OR}\;=\;e^{\left(\beta_{xy}\times 0.693\right)}$$

This is the odds ratio corresponding to doubling (ln 2 ≈ 0.693) the exposure prevalence (Burgess and Labrecque 2018).

### Sensitivity Analyses

The IV framework requires that a valid instrument is robustly associated with the exposure (“relevance”), does not share common causes with the outcome (“exchangeability”), and exclusively affects the outcome through the exposure (“exclusion restriction”; i.e., horizontal pleiotropy should be absent) (Labrecque and Swanson 2018). Violations of these (core) assumptions could invalidate the IVW estimate. To examine possible violations of the exchangeability and exclusion restriction assumptions via correlated and uncorrelated pleiotropic pathways, we searched the instruments in Phenoscanner (Yang, Sanderson, et al. 2022). We assessed the IVW model’s validity using the Cochran *Q* statistics and the mendelian randomisation (MR) Egger intercept test. The *Q* statistic quantifies horizontal pleiotropy and heterogeneity, with significant values indicating that assumptions are violated (Del Greco M et al. 2015). A nonzero MR Egger intercept points toward the presence of directional (nonmean zero) horizontal pleiotropy (Hemani et al. 2018). To avoid the IVW estimate being substantially influenced by a single or a few SNPs, which could also indicate pleiotropic effects, we performed a leave-one-out analysis. Here, 1 SNP at a time is iteratively dropped from the analysis (Burgess et al. 2019). Moreover, we analyzed the individual SNP/Wald ratio estimates for outliers. We implemented several robust IV methods as sensitivity analyses. The robust methods differ both in the way the causal effect is estimated and in the assumptions underlying these calculations (Slob and Burgess 2020). The weighted median provides a constant estimate as long as 50% of the analysis’ weight is given by valid instruments. The Robust Adjusted Profile Score (MR-RAPS) was designed to provide robust causal effect estimates in the presence of pleiotropy, weak instrument bias, and extreme outliers. This is achieved by modeling the pleiotropic effects of SNPs using a random-effects distribution. The MR-RAPS estimates are then obtained using a profile likelihood function for the causal effect and the variance of the pleiotropic effect distribution. As long as the pleiotropy is balanced (averaging to zero), this model permits all SNPs to be invalid due to pleiotropy. The employed MR pleiotropy residual sum and outlier (MR-PRESSO) method removes SNPs based on their contribution to heterogeneity from the analysis. The IVW is then used to derive the causal estimate, leveraging only the remaining genetic variants (Slob and Burgess 2020). Last, we used the constrained maximum likelihood (c-ML) approach to account for SNPs with pleiotropic effects. This method is robust to invalid IVs with uncorrelated and/or correlated pleiotropic effects and is based on the “plurality valid” assumption, which is weaker than the weighted median’s “majority valid” premise (Xue et al. 2021).

The analyses were performed using the TwoSampleMR (0.5.6), MendelianRandomization (0.7.0), and MRPRESSO (1.0) packages in R, version 4.3.0.

### Ethics

All analyses relied on publicly accessible summary statistics without any individual-level data, so no ethical approval was needed. The included GWAS were authorized by relevant local ethical review boards, and study participants provided informed consent.

## Results

### Instrument Selection

We identified 57 SNPs as genetic instruments for SjD, accounting for 1.4% of the phenotypic variation. Each of these SNPs demonstrated an *F* statistic >10, minimizing the likelihood of weak instrument bias and making any violation of the relevance assumption unlikely. Detailed characteristics of the genetic instruments can be found in Appendix Tables 1 and 2.

### Primary and Sensitivity Analyses

Table 2 presents the results of the primary analyses and complements them with the results of the sensitivity analyses. The primary IVW analyses identified significant associations between SjD and both dental caries (measured as DMFS) (β = 0.039, *P* = 6.3e-16) and periodontitis (OR = 1.033, *P* = 2.3e-05).

**Table 2.** Summary of MR Estimates for Each Dental Outcome Comparing Primary Inverse Variance Estimates and Sensitivity Methods.

	Nsnp	Method	β	Standard Error	Odds Ratio/Transformed Effect	95% Confidence Interval	*P* ^a^
DMFS index	57	IVW	0.039	0.005	0.779 surfaces	0.590–0.968	6.3e-16
		Weighted median	0.041	0.007	0.807 surfaces	0.529–1.085	1.3e-08
		RAPS	0.034	0.005	0.667 surfaces	0.492–0.843	1.0e-13
		Presso	0.039	0.004	0.779 surfaces	0.611–0.948	1.3e-12
		c-ML	0.040	0.005	0.790 surfaces	0.598–0.982	7.6e-16
Periodontitis	57	IVW	0.032	0.008	OR = 1.033	1.017–1.048	2.3e-05
		Weighted median	0.029	0.010	OR = 1.030	1.009–1.051	4.2e-03
		RAPS	0.026	0.007	OR = 1.026	1.012–1.040	1.9e-04
		Presso	0.032	0.008	OR = 1.033	1.017–1.048	8.6e-05
		c-ML	0.032	0.007	OR = 1.030	1.012–1.055	7.9e-06

c-ML, constrained maximum likelihood; DMFS, decayed, missing, and filled surfaces; IVW, inverse variance weighted; MR, mendelian randomization; Nsnp, number of single-nucleotide polymorphisms; OR, odds ratio; RAPS, Robust Adjusted Profile Score; Presso, pleiotropy residual sum and outlier.

a *P* values test the null hypothesis of no causal association between Sjögren’s disease and oral health outcomes.

The PhenoScanner search revealed previous reports of associations between genetic instruments and autoreactivity traits (see Appendix Table 3). The *Q* statistic indicated no evidence of heterogeneity, and the MR-Egger intercept test provided no support for unbalanced pleiotropy (Appendix Table 4). The robustness of our IVW estimates was further confirmed by leave-one-out analyses, which demonstrated that excluding any single SNP did not significantly alter the overall results. The observed consistency shows that no individual instrument had an excessive influence. Moreover, an analysis of individual SNP/Wald ratio estimates did not identify any leverage points, as illustrated in Appendix Figures 1 to 4. Ultimately, results derived from all robust methods applied to assess potential violations of our assumptions were consistent with our original IVW estimates, strengthening the credibility of our findings.

## Discussion

Using an IV approach, our study presents compelling evidence that not only supports the prevailing hypothesis of SjD increasing caries burden but also strengthens the argument for an elevated risk of periodontitis (Pedersen et al. 2005; Carr et al. 2012; Brito-Zerón et al. 2016). To the best of our knowledge, this is the first implementation of an IV framework to examine these specific associations and thereby enhances our understanding of the oral consequences of SjD.

Our results challenge prior studies that claimed SjD only affects caries but not periodontitis. A meta-analysis of 10 cross-sectional studies, consisting of 228 cases of SjD and 223 controls, concluded that, while markers of periodontal burden were elevated (clinical attachment loss (CAL): mean difference: 0.10; 95% CI, −0.29 to 0.49, *P* = 0.60; pocket probing depth (PPD): mean difference: 0.12; 95% CI, −0.04 to 0.28, *P* = 0.14), a significant and robust difference could only be assumed for caries (DMFS: mean difference: 4.42; 95% CI, 2.44–6.41, *P* = 0.0001) (Maarse et al. 2019). Therefore, the authors stated that SjD should not be considered a risk factor for periodontal disease. In another systematic review, encompassing 17 studies with a total of 518 individuals with SjD and 544 healthy controls, similar conclusions were drawn (de Goés Soares et al. 2018). Despite observing elevated indices of periodontal inflammation, the authors deemed the collective evidence from the studies insufficient to establish a causal relationship. It is noteworthy that both meta-analyses show a substantive overlap of the included studies, suggesting a degree of redundancy in the literature examined.

Contrarily, a recent meta-analysis that incorporated 5 studies and collectively comprised 6,929 participants supported our findings. Yang and colleagues identified a positive association between SjD and periodontitis, presenting an OR of 2.12 (95% CI, 1.43–3.17) (Yang, Pang, et al. 2022). The authors further conducted sensitivity analyses—a meta-analysis of 16 studies and a systematic review of 21 investigations, totaling 11,435 individuals—which supported this conclusion. Among the 3 meta-analyses discussed, the latter seems to provide a more transparent and comprehensive assessment of bias and heterogeneity. Furthermore, a prospective study that examined the occurrence of SjD in 135,190 patients over a follow-up of 7 years, among whom 27,041 had periodontal disease, also suggests a link between the 2 diseases (Lin et al. 2019).

Different authors draw divergent conclusions due to inherent limitations of observational studies (i.e., confounding, reverse causation) and the complex pathogenesis of the disorders under scrutiny (Fu and Kim 2021). SjD is a multifaceted condition, and early symptoms (e.g., chronic inflammation, gradual loss of salivary function) manifest up to 2 decades before the actual diagnosis is made. Thus, negative effects on the oral cavity may arise prior to the clinically reported disease onset. Moreover, SjD is associated with various comorbidities, and it remains unclear which of them are merely concurrent conditions and which are rooted in the disease’s autoreactivity (Brito-Zerón et al. 2016). Therefore, the results of traditional observational studies are likely to be distorted due to (unmeasured) confounding. This applies to both caries and periodontitis. Caries, however, is less heavily influenced by systemic factors, and xerostomia is a well-studied risk factor for tooth decay. In contrast, the influence of saliva composition and properties on the periodontium is not yet fully understood (Dawes and Wong 2019). Along with the reduced mechanical and biological functions of saliva, such as plaque reduction and antimicrobial activity, the exacerbation of periodontitis in SjD could also be rooted in the host’s altered immune response itself (Yang, Pang, et al. 2022). This potential connection to the periodontium may involve the perpetuation of the autoimmune process through an interplay between the innate and adaptive immune systems, leading to chronic B-cell stimulation, immune-complex deposition, and extranodal lymphoproliferation (Mavragani 2017). In theory, even disturbances in the neuroendocrine system could potentially serve as a plausible connection to the periodontium (Tzioufas et al. 2008). However, the precise pathomechanisms underlying the symptoms of SjD largely remain unexplored to date.

The primary strength of our study is the use of randomly allocated SNPs at conception as instrumental variables, effectively reducing confounding bias and reverse causation. This framework enables to draw causal inferences from observational data without assuming the absence of unmeasured confounding (Davies et al. 2018). Another notable advantage is found in the distinct pathogenesis of both dental traits, while the literature indicates the “true” direction of the effect from SjD on caries. Caries can thus be considered a positive control outcome (i.e., a causal relationship that, if not detected, raises concerns about the statistical power or the validity of the instruments). Given that we were able to replicate this anticipated effect, it logically follows that the tools employed in this study can also be deemed valid for detecting the impact and direction of SjD on periodontitis (Palmer et al. 2013; Burgess et al. 2019). The argument of valid IVs is further strengthened by the sensitivity analyses employed in our study, supporting the primary results across a variety of assumptions.

Nevertheless, our study has inherent limitations that need to be considered. First, the phenotypes in the SjD and periodontitis GWAS were only broadly defined. This misclassification bias may lead to imprecision of the effect estimate and might attenuate it toward the null (Clayton et al. 2023). The potential underestimation of the impact becomes apparent in comparison with the reported effect of SjD on dental caries in existing literature, which significantly exceeds the effect determined in our study (Carr et al. 2012; Maarse et al. 2019). Nonetheless, even if our effect estimates might be attenuated, they still reflect the presence and direction of a potential causal pathway. Second, the summary data used are lacking individual information, rendering subgroup analyses impossible. Third, horizontal pleiotropy cannot be ruled out completely in our IV framework, even if our sensitivity analyses provided reassurance by not indicating its presence. Last, our study focused on a population with European ancestry, and caution should be exercised when generalizing these findings to other populations.

## Conclusion

Recognizing these strengths and limitations and leveraging the fact that they differ from those of traditional observational methods, our analysis represents a valuable contribution to understanding the impacts of SjD on oral health. Nonetheless, further studies, optimally using varying designs, are necessary to elucidate the consequences of SjD more precisely. This knowledge is invaluable for future clinical guidelines, both for maintaining overall health and for oral rehabilitation in patients affected by SjD.

## Author Contributions

S.L. Reckelkamm, S.E. Baumeister, contributed to conception, design, data acquisition, analysis, and interpretation, drafted and critically revised the manuscript; Z. Alayash, contributed to conception, data analysis, critically revised the manuscript; B. Holtfreter, contributed to design, data interpretation, critically revised the manuscript; M. Nolde, contributed to conception, design, data analysis and interpretation, critically revised the manuscript. All authors gave final approval and agree to be accountable for all aspects of the work.

## Supplemental Material

sj-docx-1-jdr-10.1177_00220345231218903 – Supplemental material for Sjögren’s Disease and Oral Health: A Genetic Instrumental Variable Analysis Supplemental material, sj-docx-1-jdr-10.1177_00220345231218903 for Sjögren’s Disease and Oral Health: A Genetic Instrumental Variable Analysis by S.L. Reckelkamm, Z. Alayash, B. Holtfreter, M. Nolde and S.E. Baumeister in Journal of Dental Research

sj-docx-2-jdr-10.1177_00220345231218903 – Supplemental material for Sjögren’s Disease and Oral Health: A Genetic Instrumental Variable Analysis Supplemental material, sj-docx-2-jdr-10.1177_00220345231218903 for Sjögren’s Disease and Oral Health: A Genetic Instrumental Variable Analysis by S.L. Reckelkamm, Z. Alayash, B. Holtfreter, M. Nolde and S.E. Baumeister in Journal of Dental Research
