# Supplementary material for: Sjögren’s Disease and Oral Health: A Genetic Instrumental Variable Analysis
Source: J Dent Res. 2024 Jan 29;103(3):263–8. doi: 10.1177/00220345231218903 (PMC10900855; doi:10.1177/00220345231218903)
Supplement: sj-docx-1-jdr-10.1177_00220345231218903 – Supplemental material for Sjögren’s Disease and Oral Health: A Genetic Instrumental Variable Analysis [file sj-docx-1-jdr-10.1177_00220345231218903.docx]

## Supplementary Material

## Supplementary Table 1

Associations of single nucleotide polymorphisms with Sjögren’s Disease and DMFS index

| Estimates for Sjögren’s Disease | | | | | | | | Estimates for DMFS index | | |
| --- | --- | --- | --- | --- | --- | --- | --- | --- | --- | --- |
| SNP | EA | OA | EAF | BETA | SE | P | F | BETA | SE | P |
| rs10174238 | A | G | 0.764 | -0.200 | 0.034 | 4.7e-09 | 34.3 | -0.006 | 0.010 | 5.3e-01 |
| rs112104961 | G | T | 0.108 | 0.387 | 0.043 | 4.0e-19 | 79.9 | 0.033 | 0.012 | 5.7e-03 |
| rs113547322 | T | G | 0.269 | -0.325 | 0.035 | 2.3e-20 | 85.5 | -0.016 | 0.012 | 1.8e-01 |
| rs113858286 | G | C | 0.029 | 0.435 | 0.079 | 4.0e-08 | 30.2 | 0.031 | 0.038 | 4.2e-01 |
| rs12614 | T | C | 0.144 | -0.299 | 0.046 | 5.0e-11 | 43.2 | -0.027 | 0.013 | 4.3e-02 |
| rs12662525 | C | T | 0.193 | 0.215 | 0.036 | 3.3e-09 | 35.0 | 0.010 | 0.011 | 3.6e-01 |
| rs1281933 | T | C | 0.091 | -0.340 | 0.057 | 2.4e-09 | 35.6 | 0.003 | 0.018 | 8.8e-01 |
| rs143917797 | T | C | 0.095 | -0.336 | 0.056 | 2.1e-09 | 35.8 | -0.014 | 0.041 | 7.4e-01 |
| rs154977 | G | C | 0.728 | -0.373 | 0.031 | 1.4e-32 | 141.2 | -0.012 | 0.009 | 2.0e-01 |
| rs1610741 | G | A | 0.300 | 0.190 | 0.032 | 2.3e-09 | 35.7 | 0.013 | 0.009 | 1.4e-01 |
| rs16869677 | T | C | 0.078 | 0.356 | 0.050 | 1.4e-12 | 50.1 | 0.011 | 0.016 | 4.9e-01 |
| rs17214106 | A | G | 0.097 | 0.508 | 0.044 | 5.4e-31 | 134.0 | 0.011 | 0.015 | 4.5e-01 |
| rs2004640 | G | T | 0.477 | -0.254 | 0.030 | 1.6e-17 | 72.6 | 0.001 | 0.009 | 9.2e-01 |
| rs2071287 | T | C | 0.458 | 0.205 | 0.030 | 5.1e-12 | 47.6 | 0.008 | 0.009 | 3.7e-01 |
| rs2071627 | A | C | 0.384 | 0.248 | 0.030 | 1.6e-16 | 68.1 | 0.013 | 0.009 | 1.7e-01 |
| rs2229094 | C | T | 0.323 | -0.236 | 0.033 | 6.5e-13 | 51.7 | -0.016 | 0.010 | 1.2e-01 |
| rs2243873 | A | C | 0.494 | -0.252 | 0.030 | 2.4e-17 | 71.8 | -0.010 | 0.009 | 2.8e-01 |
| rs2253487 | A | G | 0.369 | -0.232 | 0.032 | 1.6e-13 | 54.4 | -0.005 | 0.009 | 6.1e-01 |
| rs2267647 | G | A | 0.713 | -0.231 | 0.032 | 4.0e-13 | 52.6 | -0.004 | 0.009 | 6.3e-01 |
| rs2393923 | C | T | 0.182 | 0.217 | 0.037 | 5.0e-09 | 34.2 | 0.004 | 0.010 | 7.0e-01 |
| rs2523674 | G | A | 0.366 | 0.238 | 0.030 | 3.6e-15 | 61.9 | 0.005 | 0.009 | 5.9e-01 |
| rs2523990 | G | A | 0.584 | 0.177 | 0.031 | 7.8e-09 | 33.3 | 0.000 | 0.009 | 9.8e-01 |
| rs2534664 | A | G | 0.393 | -0.209 | 0.031 | 1.4e-11 | 45.7 | 0.010 | 0.009 | 2.4e-01 |
| rs2844575 | C | T | 0.407 | -0.215 | 0.031 | 2.5e-12 | 49.1 | 0.001 | 0.009 | 9.0e-01 |
| rs2844795 | T | C | 0.516 | -0.221 | 0.030 | 1.1e-13 | 55.2 | 0.005 | 0.009 | 5.5e-01 |
| rs2858331 | G | A | 0.325 | -0.340 | 0.033 | 3.6e-25 | 107.4 | -0.024 | 0.009 | 5.7e-03 |
| rs28752924 | C | T | 0.486 | -0.249 | 0.030 | 7.5e-17 | 69.5 | -0.027 | 0.009 | 2.5e-03 |
| rs3135402 | C | A | 0.320 | -0.207 | 0.033 | 2.4e-10 | 40.1 | -0.016 | 0.009 | 7.7e-02 |
| rs34865778 | A | G | 0.161 | 0.254 | 0.038 | 3.0e-11 | 44.2 | 0.015 | 0.012 | 2.3e-01 |
| rs35366682 | C | T | 0.295 | 0.205 | 0.032 | 1.4e-10 | 41.1 | 0.010 | 0.010 | 3.2e-01 |
| rs36229731 | C | A | 0.206 | -0.359 | 0.039 | 8.8e-20 | 82.9 | -0.020 | 0.013 | 1.4e-01 |
| rs3873446 | G | A | 0.251 | -0.234 | 0.036 | 6.8e-11 | 42.6 | 0.006 | 0.011 | 5.8e-01 |
| rs394038 | A | G | 0.204 | 0.356 | 0.034 | 4.2e-25 | 107.1 | 0.048 | 0.048 | 3.2e-01 |
| rs62397630 | T | C | 0.060 | 0.362 | 0.057 | 2.3e-10 | 40.2 | 0.037 | 0.023 | 1.1e-01 |
| rs68600 | T | C | 0.608 | -0.242 | 0.030 | 8.0e-16 | 64.9 | -0.008 | 0.009 | 3.4e-01 |
| rs6903171 | C | G | 0.116 | 0.349 | 0.043 | 6.3e-16 | 65.4 | 0.018 | 0.014 | 2.0e-01 |
| rs6909620 | C | A | 0.246 | -0.230 | 0.036 | 1.8e-10 | 40.7 | -0.009 | 0.011 | 4.2e-01 |
| rs72891915 | A | G | 0.040 | 0.478 | 0.066 | 6.4e-13 | 51.7 | -0.043 | 0.047 | 3.6e-01 |
| rs764753 | T | A | 0.732 | -0.195 | 0.033 | 2.7e-09 | 35.4 | 0.004 | 0.009 | 6.7e-01 |
| rs7769527 | G | A | 0.075 | 0.359 | 0.052 | 3.7e-12 | 48.3 | 0.000 | 0.013 | 9.9e-01 |
| rs805277 | T | C | 0.386 | -0.240 | 0.031 | 1.4e-14 | 59.2 | 0.001 | 0.009 | 9.0e-01 |
| rs885948 | G | A | 0.428 | 0.164 | 0.030 | 4.1e-08 | 30.1 | 0.009 | 0.009 | 2.9e-01 |
| rs915895 | C | T | 0.237 | 0.323 | 0.033 | 1.1e-22 | 96.0 | 0.013 | 0.009 | 1.8e-01 |
| rs9261425 | C | T | 0.677 | -0.263 | 0.031 | 2.0e-17 | 72.1 | -0.016 | 0.009 | 7.5e-02 |
| rs9262131 | T | C | 0.282 | 0.273 | 0.032 | 9.5e-18 | 73.6 | 0.011 | 0.010 | 2.4e-01 |
| rs9262617 | A | G | 0.324 | -0.257 | 0.033 | 5.0e-15 | 61.2 | -0.015 | 0.009 | 1.2e-01 |
| rs9266071 | T | C | 0.582 | -0.353 | 0.029 | 2.6e-33 | 144.6 | -0.011 | 0.009 | 2.3e-01 |
| rs9266636 | G | A | 0.306 | -0.229 | 0.033 | 6.0e-12 | 47.3 | -0.014 | 0.009 | 1.3e-01 |
| rs9267092 | T | G | 0.092 | 0.748 | 0.041 | 5.2e-73 | 326.6 | 0.047 | 0.017 | 5.0e-03 |
| rs9267513 | C | A | 0.211 | -0.255 | 0.038 | 3.1e-11 | 44.1 | 0.004 | 0.011 | 7.0e-01 |
| rs9271573 | C | A | 0.583 | -0.453 | 0.029 | 2.9e-55 | 245.2 | -0.005 | 0.009 | 5.5e-01 |
| rs9273324 | T | C | 0.198 | 0.542 | 0.033 | 6.0e-60 | 266.7 | 0.023 | 0.010 | 1.9e-02 |
| rs9275601 | T | C | 0.339 | 0.299 | 0.030 | 9.0e-23 | 96.5 | 0.014 | 0.010 | 1.3e-01 |
| rs9276606 | T | A | 0.242 | 0.238 | 0.033 | 1.0e-12 | 50.8 | 0.020 | 0.010 | 5.1e-02 |
| rs9277770 | G | T | 0.621 | -0.202 | 0.030 | 2.6e-11 | 44.4 | -0.014 | 0.009 | 1.2e-01 |
| rs9467711 | C | A | 0.054 | 0.431 | 0.059 | 2.0e-13 | 54.0 | 0.042 | 0.013 | 1.1e-03 |
| rs9469586 | A | G | 0.077 | 0.310 | 0.051 | 1.6e-09 | 36.4 | 0.020 | 0.020 | 3.1e-01 |

EA, effect allele. OA, other allele. EAF, effect allele frequency. BETA, beta regression coefficient. SE, standard error. P, P value. F, F statistic.

## Supplementary Table 2

Associations of single nucleotide polymorphisms with Sjögren’s Disease and periodontitis

|  | Estimates for Sjögren’s Disease | | | | | | | Estimates for periodontitis | | |
| --- | --- | --- | --- | --- | --- | --- | --- | --- | --- | --- |
| SNP | EA | OA | EAF | BETA | SE | P | F | BETA | SE | P |
| rs10174238 | A | G | 0.764 | -0.200 | 0.034 | 4.7e-09 | 34.3 | -0.047 | 0.018 | 1.1e-02 |
| rs112104961 | G | T | 0.108 | 0.387 | 0.043 | 4.0e-19 | 79.9 | 0.011 | 0.030 | 7.1e-01 |
| rs113547322 | T | G | 0.269 | -0.325 | 0.035 | 2.3e-20 | 85.5 | -0.030 | 0.029 | 3.0e-01 |
| rs113858286 | G | C | 0.029 | 0.435 | 0.079 | 4.0e-08 | 30.2 | -0.027 | 0.072 | 7.1e-01 |
| rs12614 | T | C | 0.144 | -0.299 | 0.046 | 5.0e-11 | 43.2 | -0.046 | 0.025 | 6.8e-02 |
| rs12662525 | C | T | 0.193 | 0.215 | 0.036 | 3.3e-09 | 35.0 | 0.008 | 0.018 | 6.6e-01 |
| rs1281933 | T | C | 0.091 | -0.340 | 0.057 | 2.4e-09 | 35.6 | -0.037 | 0.046 | 4.3e-01 |
| rs143917797 | T | C | 0.095 | -0.336 | 0.056 | 2.1e-09 | 35.8 | -0.276 | 0.121 | 2.3e-02 |
| rs154977 | G | C | 0.728 | -0.373 | 0.031 | 1.4e-32 | 141.2 | -0.006 | 0.018 | 7.3e-01 |
| rs1610741 | G | A | 0.300 | 0.190 | 0.032 | 2.3e-09 | 35.7 | 0.009 | 0.017 | 6.1e-01 |
| rs16869677 | T | C | 0.078 | 0.356 | 0.050 | 1.4e-12 | 50.1 | -0.006 | 0.028 | 8.4e-01 |
| rs17214106 | A | G | 0.097 | 0.508 | 0.044 | 5.4e-31 | 134.0 | 0.036 | 0.026 | 1.6e-01 |
| rs2004640 | G | T | 0.477 | -0.254 | 0.030 | 1.6e-17 | 72.6 | -0.036 | 0.016 | 2.3e-02 |
| rs2071287 | T | C | 0.458 | 0.205 | 0.030 | 5.1e-12 | 47.6 | 0.025 | 0.019 | 1.8e-01 |
| rs2071627 | A | C | 0.384 | 0.248 | 0.030 | 1.6e-16 | 68.1 | 0.017 | 0.016 | 2.9e-01 |
| rs2229094 | C | T | 0.323 | -0.236 | 0.033 | 6.5e-13 | 51.7 | 0.014 | 0.017 | 4.4e-01 |
| rs2243873 | A | C | 0.494 | -0.252 | 0.030 | 2.4e-17 | 71.8 | -0.001 | 0.016 | 9.5e-01 |
| rs2253487 | A | G | 0.369 | -0.232 | 0.032 | 1.6e-13 | 54.4 | 0.005 | 0.022 | 8.2e-01 |
| rs2267647 | G | A | 0.713 | -0.231 | 0.032 | 4.0e-13 | 52.6 | -0.035 | 0.016 | 2.9e-02 |
| rs2393923 | C | T | 0.182 | 0.217 | 0.037 | 5.0e-09 | 34.2 | 0.016 | 0.017 | 3.5e-01 |
| rs2523674 | G | A | 0.366 | 0.238 | 0.030 | 3.6e-15 | 61.9 | -0.010 | 0.022 | 6.5e-01 |
| rs2523990 | G | A | 0.584 | 0.177 | 0.031 | 7.8e-09 | 33.3 | 0.009 | 0.022 | 6.9e-01 |
| rs2534664 | A | G | 0.393 | -0.209 | 0.031 | 1.4e-11 | 45.7 | -0.009 | 0.016 | 5.4e-01 |
| rs2844575 | C | T | 0.407 | -0.215 | 0.031 | 2.5e-12 | 49.1 | 0.039 | 0.022 | 7.4e-02 |
| rs2844795 | T | C | 0.516 | -0.221 | 0.030 | 1.1e-13 | 55.2 | 0.001 | 0.022 | 9.6e-01 |
| rs2858331 | G | A | 0.325 | -0.340 | 0.033 | 3.6e-25 | 107.4 | -0.014 | 0.022 | 5.2e-01 |
| rs28752924 | C | T | 0.486 | -0.249 | 0.030 | 7.5e-17 | 69.5 | 0.013 | 0.022 | 5.8e-01 |
| rs3135402 | C | A | 0.320 | -0.207 | 0.033 | 2.4e-10 | 40.1 | 0.009 | 0.023 | 7.0e-01 |
| rs34865778 | A | G | 0.161 | 0.254 | 0.038 | 3.0e-11 | 44.2 | 0.012 | 0.022 | 6.0e-01 |
| rs35366682 | C | T | 0.295 | 0.205 | 0.032 | 1.4e-10 | 41.1 | 0.035 | 0.024 | 1.5e-01 |
| rs36229731 | C | A | 0.206 | -0.359 | 0.039 | 8.8e-20 | 82.9 | -0.083 | 0.033 | 1.1e-02 |
| rs3873446 | G | A | 0.251 | -0.234 | 0.036 | 6.8e-11 | 42.6 | -0.025 | 0.027 | 3.5e-01 |
| rs394038 | A | G | 0.204 | 0.356 | 0.034 | 4.2e-25 | 107.1 | 0.128 | 0.115 | 2.7e-01 |
| rs62397630 | T | C | 0.060 | 0.362 | 0.057 | 2.3e-10 | 40.2 | -0.059 | 0.041 | 1.6e-01 |
| rs68600 | T | C | 0.608 | -0.242 | 0.030 | 8.0e-16 | 64.9 | -0.027 | 0.017 | 9.8e-02 |
| rs6903171 | C | G | 0.116 | 0.349 | 0.043 | 6.3e-16 | 65.4 | 0.033 | 0.027 | 2.2e-01 |
| rs6909620 | C | A | 0.246 | -0.230 | 0.036 | 1.8e-10 | 40.7 | -0.024 | 0.027 | 3.8e-01 |
| rs72891915 | A | G | 0.040 | 0.478 | 0.066 | 6.4e-13 | 51.7 | 0.032 | 0.082 | 6.9e-01 |
| rs764753 | T | A | 0.732 | -0.195 | 0.033 | 2.7e-09 | 35.4 | -0.001 | 0.017 | 9.7e-01 |
| rs7769527 | G | A | 0.075 | 0.359 | 0.052 | 3.7e-12 | 48.3 | -0.004 | 0.024 | 8.6e-01 |
| rs805277 | T | C | 0.386 | -0.240 | 0.031 | 1.4e-14 | 59.2 | -0.006 | 0.018 | 7.5e-01 |
| rs885948 | G | A | 0.428 | 0.164 | 0.030 | 4.1e-08 | 30.1 | 0.001 | 0.018 | 9.4e-01 |
| rs915895 | C | T | 0.237 | 0.323 | 0.033 | 1.1e-22 | 96.0 | 0.026 | 0.023 | 2.7e-01 |
| rs9261425 | C | T | 0.677 | -0.263 | 0.031 | 2.0e-17 | 72.1 | -0.006 | 0.022 | 7.8e-01 |
| rs9262131 | T | C | 0.282 | 0.273 | 0.032 | 9.5e-18 | 73.6 | 0.040 | 0.024 | 8.7e-02 |
| rs9262617 | A | G | 0.324 | -0.257 | 0.033 | 5.0e-15 | 61.2 | 0.025 | 0.023 | 2.7e-01 |
| rs9266071 | T | C | 0.582 | -0.353 | 0.029 | 2.6e-33 | 144.6 | 0.026 | 0.022 | 2.3e-01 |
| rs9266636 | G | A | 0.306 | -0.229 | 0.033 | 6.0e-12 | 47.3 | 0.011 | 0.022 | 6.4e-01 |
| rs9267092 | T | G | 0.092 | 0.748 | 0.041 | 5.2e-73 | 326.6 | 0.029 | 0.042 | 4.8e-01 |
| rs9267513 | C | A | 0.211 | -0.255 | 0.038 | 3.1e-11 | 44.1 | -0.048 | 0.021 | 2.4e-02 |
| rs9271573 | C | A | 0.583 | -0.453 | 0.029 | 2.9e-55 | 245.2 | -0.028 | 0.022 | 2.0e-01 |
| rs9273324 | T | C | 0.198 | 0.542 | 0.033 | 6.0e-60 | 266.7 | 0.062 | 0.025 | 1.2e-02 |
| rs9275601 | T | C | 0.339 | 0.299 | 0.030 | 9.0e-23 | 96.5 | 0.004 | 0.024 | 8.6e-01 |
| rs9276606 | T | A | 0.242 | 0.238 | 0.033 | 1.0e-12 | 50.8 | -0.012 | 0.026 | 6.3e-01 |
| rs9277770 | G | T | 0.621 | -0.202 | 0.030 | 2.6e-11 | 44.4 | 0.031 | 0.018 | 9.5e-02 |
| rs9467711 | C | A | 0.054 | 0.431 | 0.059 | 2.0e-13 | 54.0 | 0.014 | 0.024 | 5.5e-01 |
| rs9469586 | A | G | 0.077 | 0.310 | 0.051 | 1.6e-09 | 36.4 | 0.021 | 0.036 | 5.6e-01 |

EA, effect allele. OA, other allele. EAF, effect allele frequency. BETA, beta regression coefficient. SE, standard error. P, P value. F, F statistic.

## **Supplementary Table 3**

Association (P<5x10-8) of the SNPs used as instruments in the analyses with phenotypic traits in PhenoScanner (accessed on 2023/05/22)

| Phenotypic association | Times reported |
| --- | --- |
| Self-reported malabsorption or coeliac disease | 42 |
| Rheumatoid arthritis | 38 |
| Intestinal malabsorption | 35 |
| White blood cell count | 32 |
| Primary sclerosing cholangitis | 30 |
| Lymphocyte count | 28 |
| Sum eosinophil basophil counts | 26 |
| Eosinophil count | 25 |
| IgA deficiency | 24 |
| Myeloid white cell count | 24 |
| Self-reported hyperthyroidism or thyrotoxicosis | 24 |
| Granulocyte count | 23 |
| Monocyte count | 22 |
| Sitting height | 21 |
| Sum basophil neutrophil counts | 20 |
| Sum neutrophil eosinophil counts | 20 |

Phenoscanner search with genomewide significance and r2 of 0.8. Counted were the number of times a trait was associated with a used SNP. Results are listed when at least n = 20.

## **Supplementary Table 4**

Heterogeneity of Wald ratios and MR-Egger test for directional pleiotropy

| Outcome | Heterogeneity | | |
| --- | --- | --- | --- |
|  | Q | Degrees of Freedom | P-value |
| DMFS index | 44.388 | 56 | 0.869 |
| Periodontitis | 61.603 | 56 | 0.283 |
|  | MR-Egger test for directional pleiotropy | | |
|  | Intercept | Standard error | P-value |
| DMFS index | -0.005 | 0.004 | 0.244 |
| Periodontitis | -0.007 | 0.010 | 0.504 |

Abbreviations: DMFS, Decayed, Missing, and Filled Surfaces

## **Supplementary Figure 1**

Inverse variance weighted estimates in leave-one-out analysis for the effect of Sjögren’s Disease on DMFS index


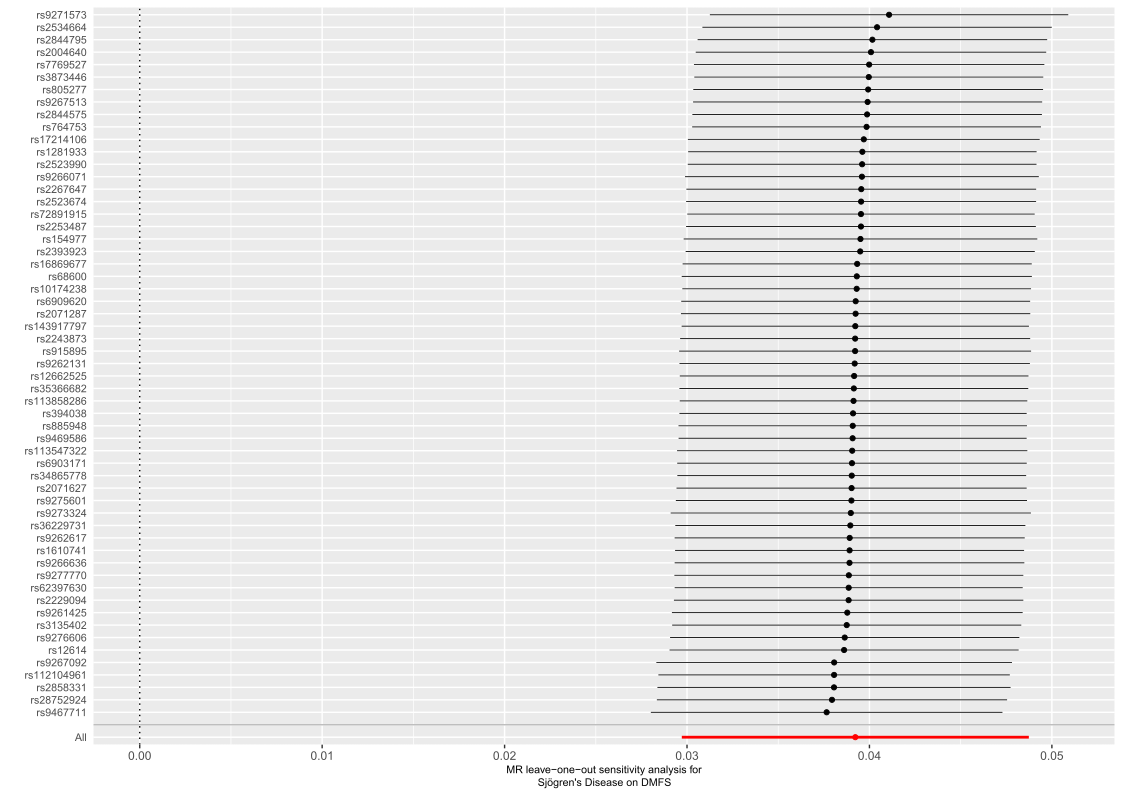


Abbreviations: SNP, single-nucleotide polymorphism; DMFS, Decayed, Missing, and Filled Surfaces

## **Supplementary Figure 2**

Inverse variance weighted estimates in leave-one-out analysis for the effect of Sjögren’s Disease on periodontitis


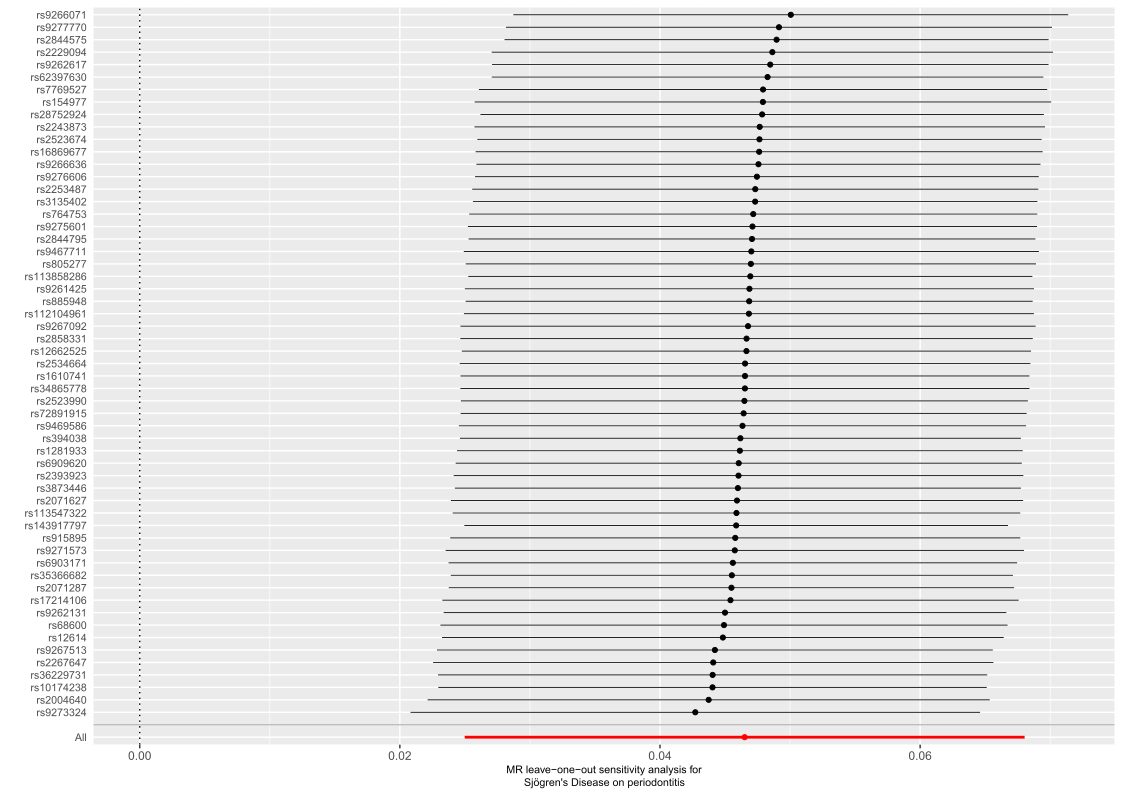


Abbreviations: SNP, single-nucleotide polymorphism

## **Supplementary Figure 3**

Forrest plot of single SNP Wald ratio estimates for the effect of Sjögren’s Disease on the Decayed, Missing, and Filled Surfaces index


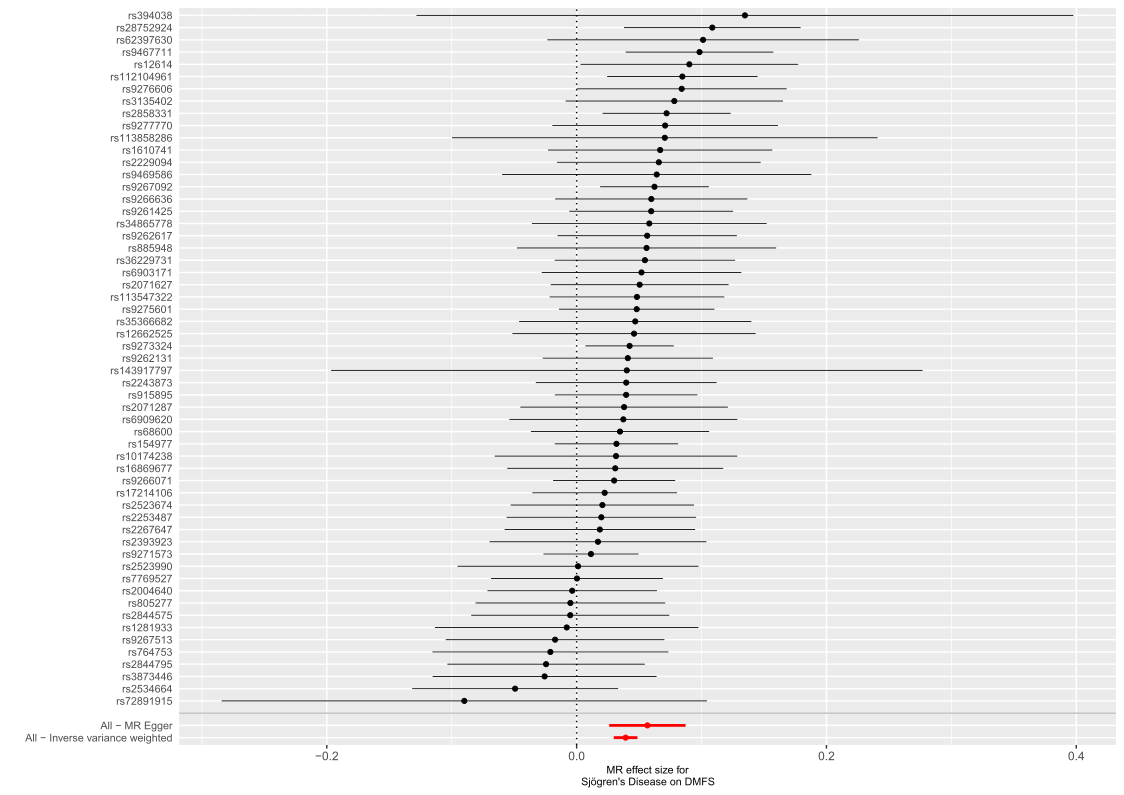


Abbreviations: SNP, single-nucleotide polymorphism; DMFS, Decayed, Missing, and Filled Surfaces

## **Supplementary Figure 4**

Forrest plot of single SNP Wald ratio estimates for the effect of Sjögren’s Disease on periodontitis


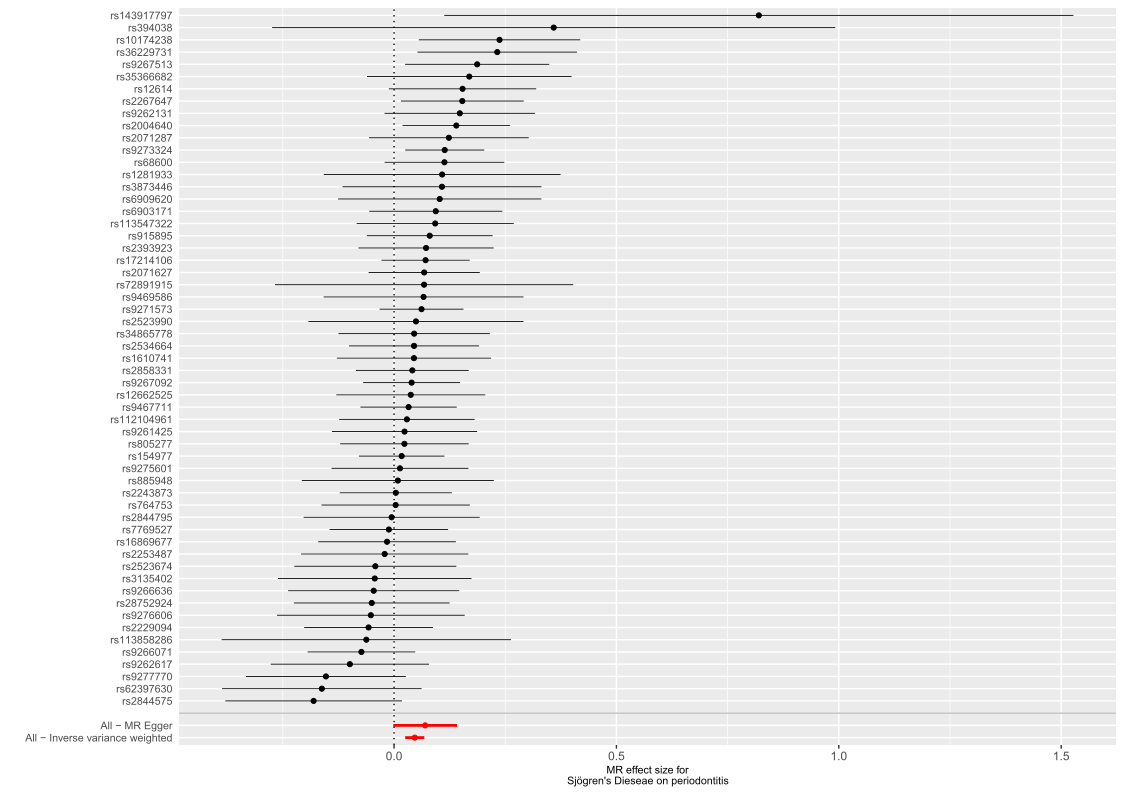


SNP, single-nucleotide polymorphism
